# Tuning the Sensitivity of Fluorescent Porphyrin Dimers to Viscosity and Temperature

**DOI:** 10.1002/chem.201700740

**Published:** 2017-06-13

**Authors:** Aurimas Vyšniauskas, Dong Ding, Maryam Qurashi, Igor Boczarow, Milan Balaz, Harry L. Anderson, Marina K. Kuimova

**Affiliations:** ^1^ Chemistry Department Imperial College London Exhibition Road London SW7 2AZ UK; ^2^ Chemistry Research Laboratory Department of Chemistry University of Oxford Oxford OX1 3TA UK; ^3^ Present address: Underwood International College, Integrated Science and Engineering Division Yonsei University Seoul 03722 Republic of Korea

**Keywords:** fluorescence, microscopic viscosity, molecular rotors, porphyrinoids, temperature sensors

## Abstract

Conjugated porphyrin dimers have emerged as versatile viscosity‐sensitive fluorophores that are suitable for quantitative measurements of microscopic viscosity by ratiometric and fluorescence lifetime‐based methods, in a concentration‐independent manner. Here, we investigate the effect of extended conjugation in a porphyrin‐dimer structure on their ability to sense viscosity and temperature. We show that the sensitivity of the fluorescence lifetime to temperature is a unique property of only a few porphyrin dimers.

## Introduction

Microscopic viscosity is an important characteristic of a heterogeneous material and can have wide implications for the macroscopic properties of an object. Ultimately, microviscosity defines viscoelastic properties of a heterogeneous material and governs diffusion rates of molecules within. Molecular rotors are a class of viscosity‐sensitive fluorophores that hold great promise for microviscosity measurements, applicable to biological and materials sciences.^1^, ^2^ Typically, fluorescence sensitivity to viscosity in molecular rotors arises from a fluorescent‐to‐“dark” state transition that is linked to a change in the conformation of a fluorophore and is therefore strongly affected by the viscosity of the environment. Furthermore, it has been demonstrated that spectral ratiometric viscosity probes^3^ or fluorescence lifetime‐based approaches^4^ make it possible to measure viscosity in a concentration‐independent manner, which is particularly useful in heterogeneous systems in which the local concentration of a fluorophore is unknown.

A particular advantage of molecular rotors over other microviscosity measurement techniques such as fluorescence correlation spectroscopy (FCS),^5^, ^6^ fluorescence recovery after photobleaching (FRAP)^7^, ^8^ or single particle tracking (SPT)^9^, ^10^ is their ability to produce viscosity images within relatively short acquisition times instead of single‐point measurements available with most alternative techniques. This advantage led to wide‐spread use of molecular rotors for dynamic microviscosity measurements in live mammalian and bacterial cells,^4^, ^11^, ^12^ as well as in model biological systems.^13^, ^14^, ^15^, ^16^

**Scheme 1:**
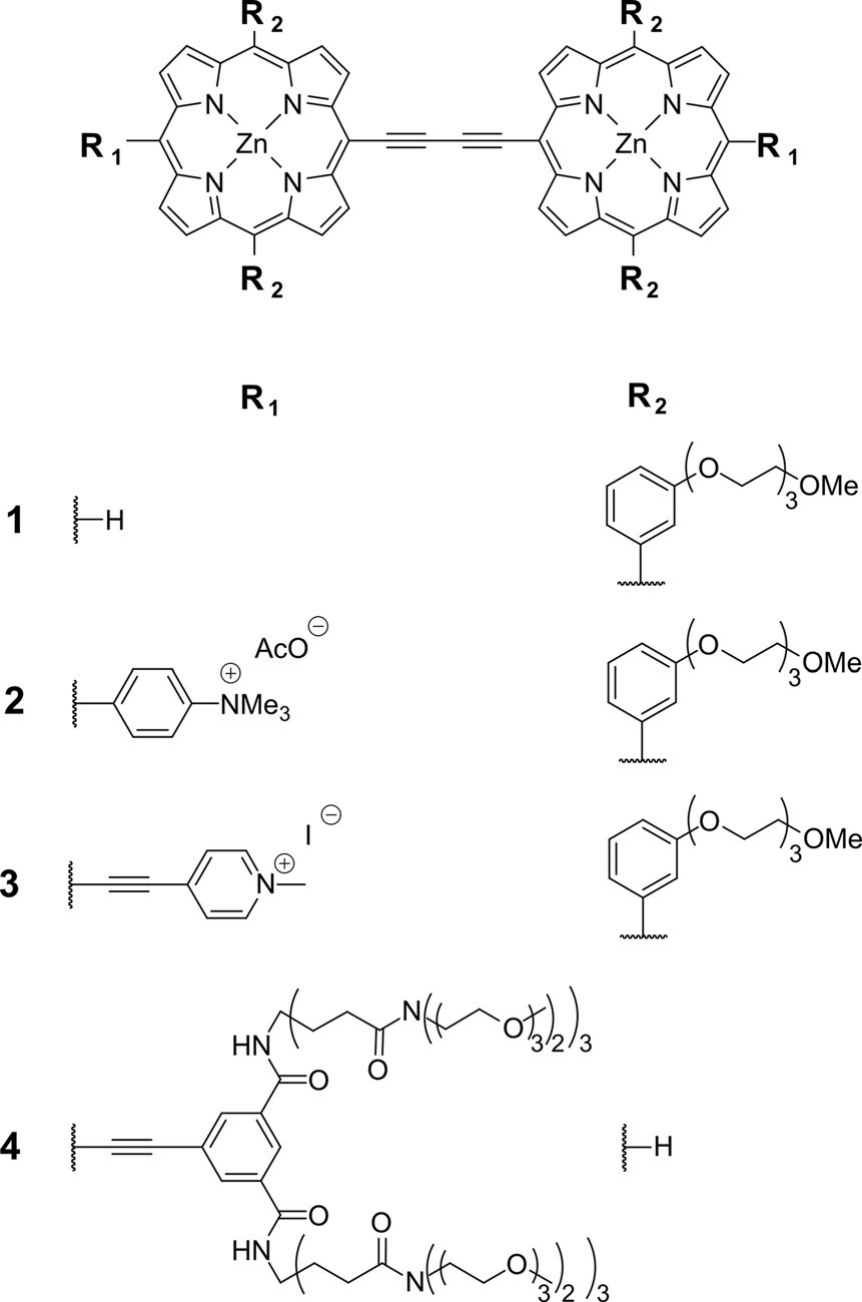
Molecular structures of porphyrin dimers **1**, **2**, **3** and **4**.

A number of conjugated porphyrin dimers have been reported as promising viscosity‐sensitive molecular rotors that are able to sense viscosity by both ratiometric and lifetime‐based methods.^17^, ^18^, ^19^ Additionally, a porphyrin dimer **2** (Scheme 1) was reported to simultaneously sense both viscosity and temperature by using combined ratiometric and lifetime‐based approaches, which makes it the only reported sensor capable of simultaneous viscosity‐temperature measurements. Here, we investigate a range of hydrophobic and hydrophilic porphyrin dimers (Scheme 1) with various degrees of conjugation to provide further insight into the photophysical behaviour of this fascinating class of fluorophores. In particular, we examine the ability of the selected dimers to simultaneously measure viscosity and temperature.

## Results and Discussion

**Figure 1:**
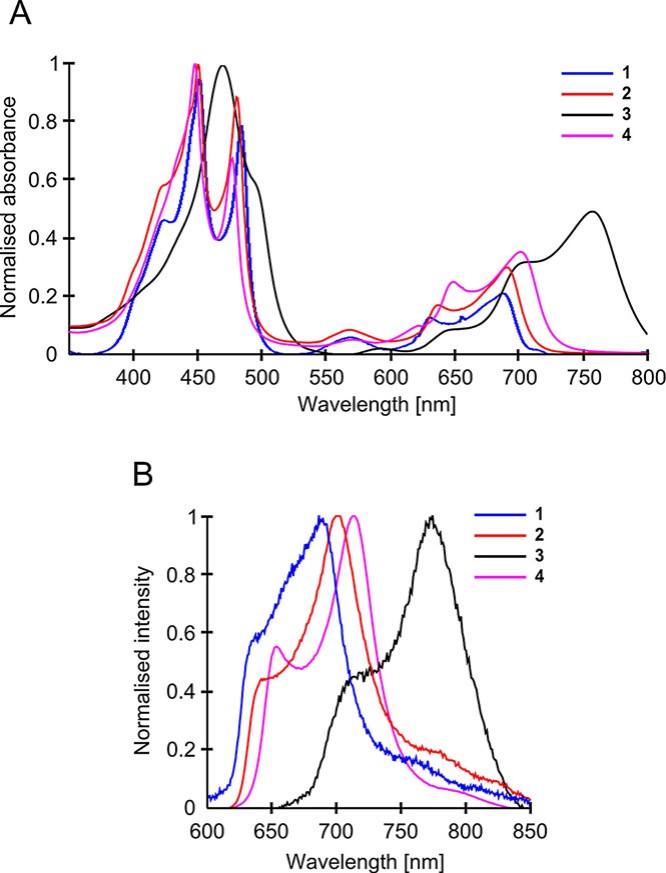
A) Absorption spectra of **1** in toluene and **2**, **3** and **4** in methanol. B) Fluorescence spectra of **1** in 70 % castor oil/toluene, **2** in 50 % glycerol/methanol, **3** and **4** in 60 % glycerol/methanol. Excitation wavelengths were 450 nm (**1**, **4**), 453 nm (**2**) and 473 nm (**3**).

The absorption and fluorescence spectra of dimers **1**–**4** are shown in Figure 1. In agreement with the literature,^20^ absorption spectra of all dimers contain peaks that can be grouped into B, also called Soret, (400–500 nm) and Q (600–800 nm) bands. It should be noted that the absorption spectra of dimers **1**, **2** and **4** have similar shapes with maxima at similar wavelengths, while the bands for dimer **3** are significantly red‐shifted, consistent with an increased degree of conjugation due to the electron‐accepting pyridinium groups. Compared to the other dimers, the B band of **3** is also significantly broadened, which suggests that a wider distribution of conformers is present. Surprisingly, the spectrum of **4** is similar to the spectra of dimers **1** and **2** in spite of the structural similarity to **3**, in which the triple bonds significantly extend conjugation.

The emission spectra of all dimers, when excited in the maximum of the B absorption band, are characterised by two distinct peaks, which give rise to the viscosity‐sensitive behaviour of the dimers (Figure 1 B). The fluorescence spectrum of dimer **3** is significantly red‐shifted compared to **1**, **2** and **4**.

The photophysical behaviour of a conjugated porphyrin dimer similar to **3** and **4** was first described by Winters et al.^21^ It was reported that the two distinct fluorescence bands arise due to a contribution of twisted and planar conformers of the dimer, formed as a result of rotation around the central butadiyne bridge. Therefore, the viscosity sensitivity of the fluorescence of porphyrin dimers^17^ arises from the interplay between the twisted and the planar conformations. These conformations appear to interconvert freely in the ground state,^22^ but display a viscosity‐dependent interconversion rate in the excited state via a barrier between the higher lying twisted conformer and a lower energy planar conformer.^23^ It follows that when excited at a higher‐energy peak in the B band, viscosity‐dependent two‐band fluorescence spectra^17^, ^18^ or time‐resolved fluorescence decays^18^ can be detected. Instead of operating via the twisted intermolecular charge transfer (TICT) mechanism, the force driving the change of excited state conformation of porphyrin dimers is a stronger cumulenic character of the central butadiyne bridge, which stabilises the planar conformation. No charge transfer takes place in either the twisted or the planar conformer, that is, both HOMO and LUMO are evenly delocalised over the whole dimer.^21^, ^22^

The excitation spectra (Figure S1 in the Supporting Information) reveal distinct wavelength regions in which pure “planar” and pure “twisted” excited state conformers can be excited, with excitation maxima independent of the solvent viscosity.^24^ While only a small overlap in twisted and planar excitation spectra was recorded for **1**, **2** and **4,** the spectra for **3** show a significant overlap (see Figure S1). Hence, for **3**, both conformers are simultaneously excited at all emission wavelengths.

**Figure 2:**
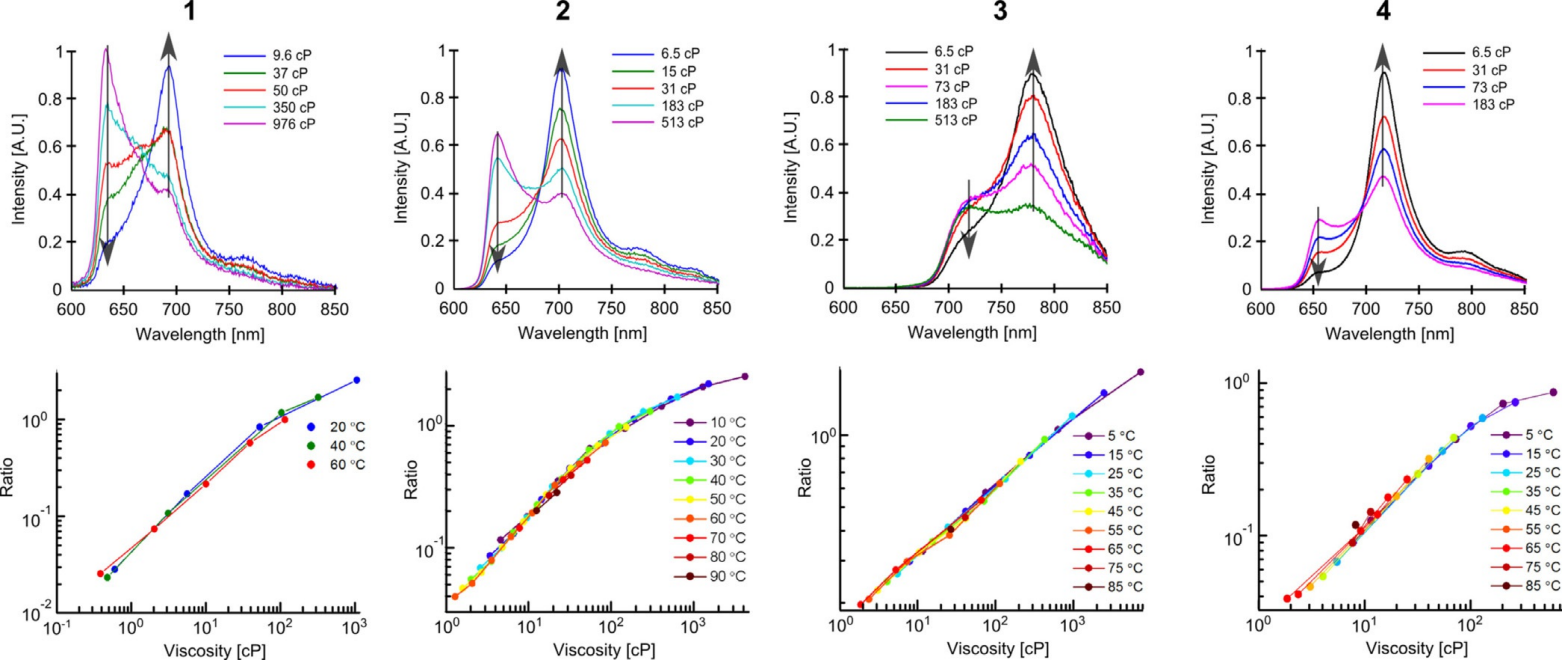
Top row: Fluorescence spectra of dimer **1** in castor oil/toluene and dimers **2**, **3**, **4** in glycerol/methanol at 20 °C. Changes in peak intensities with increasing viscosity are shown by black arrows. Viscosities of the mixtures are shown in the legend. Bottom row: Ratios of the “twisted” versus the “planar” peaks for all dimers in different viscosity mixtures recorded at a range of temperatures. Castor oil/toluene was used for **1**, glycerol/methanol for **2**, **3** and **4**. The excitation wavelengths were 450 nm (**1**, **4**), 453 nm (**2**) and 473 nm (**3**). Data for dimer **2** is reproduced from refs. 18 and 25 with permission from the Royal Society of Chemistry and the PCCP Owner Societies.

We have examined the viscosity sensitivity of dimers **1**–**4** by measuring their fluorescence spectra in mixtures of varied viscosity (Figure 2, top row). Fluorescence spectra of all dimers were obtained following excitation at the absorption maxima corresponding to the twisted conformation absorption to populate a higher‐lying twisted excited state, which leads to viscosity‐sensitive behaviour. For **1**, **2** and **4,** the excitation into the red‐edge of the B band results in an almost exclusive emission from the planar state (Figure S2), which is not useful for viscosity determination.

Due to the hydrophobic nature of **1**, the mixtures of castor oil and toluene were selected for its calibration, while glycerol and methanol mixtures were chosen for more hydrophilic derivatives **2**, **3** and **4**. In addition to varying the composition of the mixture, we also varied the temperature. Thus, an increase in the percentage of castor oil or glycerol leads to a higher viscosity and an increase in temperature leads to a lower viscosity. By independently varying both temperature and the composition of the mixtures, we were able i) to cover the widest possible range of viscosities for the purpose of calibration and ii) to examine the effect of temperature on the fluorescence properties of all dimers. While the effect of temperature on the photophysical properties of **2** was previously examined,^25^ the effect of temperature on dimers **1, 3** and **4** was studied here for the first time.

As seen from Figure 2, the emission spectra of all four dimers exhibit significant sensitivity to viscosity. Since the excitation spectral maxima were viscosity‐independent (Figure S1), the viscosity sensitivity of porphyrin dimers is not affected by conformational equilibria in the ground state as observed in so‐called “fluorescent flippers”.^26^, ^27^ As expected, the emission peak corresponding to twisted conformers shows an increased intensity at increased viscosity, whereas the intensity of the peaks due to planar conformers decreases. This response of the fluorescence spectra of the dimers to viscosity allows ratiometric calibration^3^, ^17^, ^28^, by taking the intensity ratio of the twisted peak over the planar peak in a concentration‐ independent manner.

Since we measured the ratios of two dimer peaks in a series of solvent mixtures of different solvent compositions as well as at different temperatures, we were able to verify whether the temperature can affect the fluorescence ratio of the dimers directly and not only through changing the viscosity of the mixture. The data for the overall ratiometric calibration of all dimers are shown in Figure 2 (bottom row). It is clear that the twisted/planar ratios of all dimers are sensitive to viscosity only and not to temperature, that is, the twisted/planar ratio remains the same in mixtures of identical viscosity even at different temperatures. It follows that the ratiometric spectral detection from all dimers investigated here is suitable for temperature‐independent viscosity measurements, which is a desirable characteristic for a molecular rotor. In contrast, absolute fluorescence quantum yields of the dimers could be viscosity, temperature and excitation wavelength dependent. While the quantum yields cannot be used for calibration purposes, it is useful to note that we have previously determined *Φ*_f_ for dimer **3** in non‐viscous methanol to be ten times smaller than *Φ*_f_ for **1** and **2**.^29^ Hence, the emission recorded for dimer **3** is overall somewhat dimmer than for the rest, which can complicate viscosity detection with **3** at low concentrations.

The calibration curves recorded for all dimers in the range 1–1000 cP allow us to compare the sensitivity of **1**–**4** to viscosity by the ratiometric method. The viscosity change over three orders of magnitude leads to a ratio increase by ≈65× for **1**, by ≈50× for **2**, by ≈8× for **3** and by ≈15× for **4**. Thus, dimers **1** and **2** are the most sensitive ratiometric sensors for viscosity in hydrophobic and hydrophilic environments, respectively. It should be noted that, unlike other dimers, the ratiometric calibration for dimer **3** in log–log coordinates remains linear in the whole viscosity range studied, and the values do not start to asymptotically approach the limiting values, as seen for **1**, **2** and **4** (Figure 2). However, the dynamic range for the ratiometric calibration for this dimer is somewhat smaller, which means that there are no advantages in sensitivity to changes in viscosity despite the linearity of the plot.

**Figure 3:**
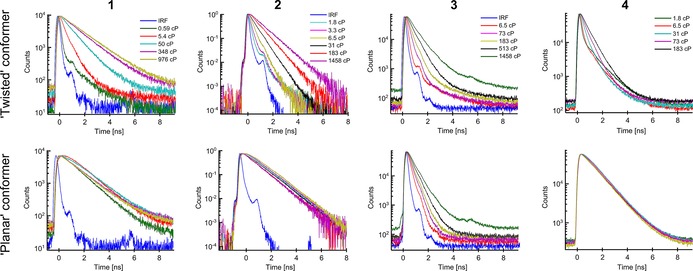
Fluorescence decays of the “twisted” and the “planar” conformers of dimers **1**, **2**, **3** and **4**. Fluorescence was excited at 450 nm (**1**, **4**), 453 nm (**2**) and 473 nm (**3**). The detection wavelengths were 630 nm and 700 nm (**1**), 640 nm and 700 nm (**2**), 710 nm and 780 nm (**3**), 650 nm and 710 nm (**4**), for the “twisted” and the “planar” conformers, respectively, with a 10 nm bandwidth detection. The data for dimer **2** (normalised decays) is reproduced from ref. 18 with permission from the PCCP Owner Societies.

We have previously demonstrated that dimer **2** belongs to a small group of molecular rotors that can report on viscosity by both ratiometric and lifetime‐based measurements.^18^ Such a dual sensing approach is beneficial, since it allows us to independently verify the viscosity read‐out using two independent measurements.^18^, ^30^ Here, we tested whether the dimers **1**, **3** and **4** also have viscosity‐sensitive fluorescence decays. Figure 3 compares time‐resolved fluorescence decays recorded in the “twisted” and the “planar” emission channels for all dimers. It is clear that the time‐resolved decays originating from high energy fluorescence bands, assigned to twisted conformers of each dimer, get progressively longer with increasing viscosity. These data confirm that all dimers act as lifetime‐based sensors for viscosity. The sensitivity of dimers **3** and **4** is slightly lower compared to dimers **1** and **2**, similar to the ratiometric calibrations (Figure 2).

Although the “twisted” time‐resolved decays have a pronounced sensitivity to viscosity, the decays of the “planar” conformers of dimers **1**, **2**, and **4** were largely viscosity‐independent (Figure 3). A small difference is an increase in a viscosity‐dependent rise‐time due to a slower twisted‐to‐planar conversion at higher viscosities. In contrast, the time‐resolved decays of the planar conformer of **3** show a strong dependence on viscosity, indicating that the initially excited population of the planar conformer of **3** decays through viscosity‐mediated conversion into some other species. This viscosity dependence of the planar conformer of **3** is an advantage over other dimers, since it means that **3** displays a viscosity‐dependent behaviour at all emission wavelengths, including extremely red‐shifted wavelengths larger than 750 nm. This allows full use of all fluorescent photons emitted by **3** for viscosity measurements in the tissue optical window.^31^ Summed up, fluorescence decays measured over the whole fluorescence spectrum of dimer **3** at different viscosities are shown in Figure S4 (Supporting Information). The viscosity dependence is clear and intensity‐weighted mean lifetimes of these decays show a marked increase with viscosity (Figure S4 B).

The unusual sensitivity of the time‐resolved fluorescence decays of the planar conformer of **3** suggested that this dimer should be further examined as a viscosity probe following two‐photon excitation. Porphyrin dimers were previously shown to possess one of the highest two‐photon absorption (TPA) cross‐sections, which makes them desirable sensors for use with two‐photon excitation.^29^, ^32^, ^33^ One of the reasons for these exceptionally high values of TPA cross‐sections is the efficient conjugation over the whole length of the molecule.^34^, ^35^ It follows that the viscosity‐insensitive planar conformer is a much better two‐photon absorber than the twisted conformation,^36^ due to a broken conjugation in the latter. This means that two‐photon excitation of the dimer should produce an excess of a planar conformer, leading to low twisted‐to‐planar ratios and viscosity‐insensitive fluorescence lifetimes, as was previously shown for **2**.^18^

The unexpected sensitivity of the planar conformer of **3** to viscosity could help to circumvent this problem. We have recorded the time‐resolved fluorescence decays of **3** following two‐photon excitation at 900 nm (Figure S3). Unfortunately, the resulting decays are characterised by a short lifetime and are insensitive to viscosity. Thus, it appears that, in contrast to one‐photon excitation, two‐photon excitation of **3** creates a subpopulation of conformations with viscosity‐independent decays and deactivate very efficiently to the ground state, even in solvents of high viscosity. We hypothesise that a wide range of slightly different planar conformers (some of them not in full conjugation) can be excited via one‐photon excitation; a viscosity‐mediated rotation of phenyl rings at both ends of the dimer can, for example, contribute to viscosity‐ dependent deactivation of the planar conformer following one‐photon excitation. As such, it appears that a viscosity‐sensitive behaviour cannot be achieved following two‐photon excitation, even for **3**.

**Figure 4:**
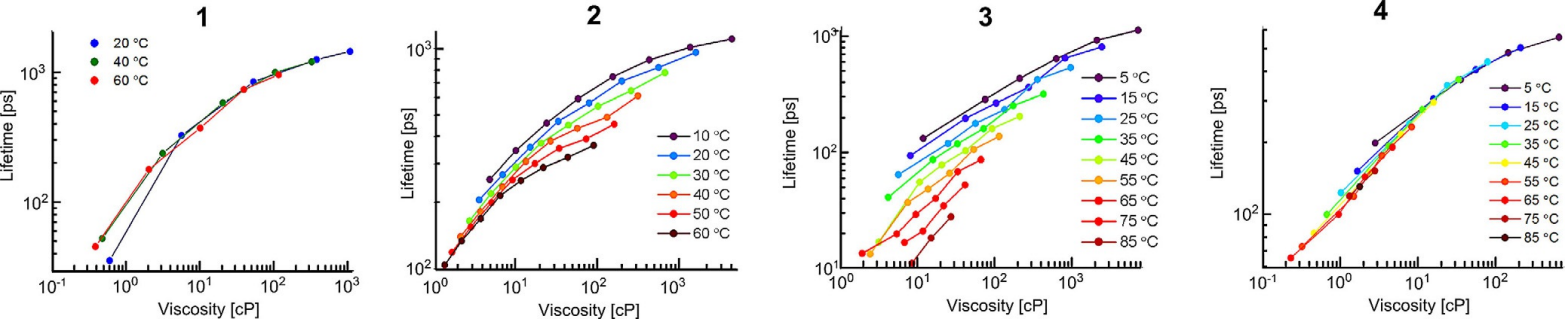
Fitted lifetimes of the “twisted” conformers of all dimers at varying viscosity in castor oil/toluene (**1**) or glycerol/methanol (**2**, **3**, **4**) at a range of temperatures. Detection wavelengths were 630 nm (**1**), 640 nm (**2**), 710 nm (**3**) and 650 nm (**4**) with a 10 nm detection bandwidth. Fluorescence was excited at 450 nm (**1**, **4**), 453 nm (**2**) and 473 nm (**3**).

We have previously shown that the lifetime of the twisted conformer of dimer **2** is sensitive to both viscosity and temperature. We used the combination of lifetime and ratiometric acquisition for measuring viscosity and temperature simultaneously.^25^ This makes **2** the only currently available probe capable of such measurements. Here, we have tested the temperature sensitivity of fluorescence decays of **1**, **3** and **4** (Figure 4). As with the ratiometric measurements for the dimers, the variation in viscosity was achieved by varying both the composition and the temperature of the calibration mixtures.

Similar to the ratiometric calibrations of all dimers, the fitted lifetimes of dimers **1** and **4** do not show any temperature dependence. Therefore, dimers **1** and **4** are suitable as dual‐mode ratio/lifetime viscosity sensors for non‐polar environments (**1**) and for moderate or high polarity environments (**4**). In contrast, the lifetime of dimer **3** shows both temperature and viscosity dependence, similarly to dimer **2**, making it also a dual viscosity and temperature sensor.

A method for simultaneous sensing of viscosity and temperature using both ratiometric and lifetime data for the porphyrin dimers is described below.

**Figure 5:**
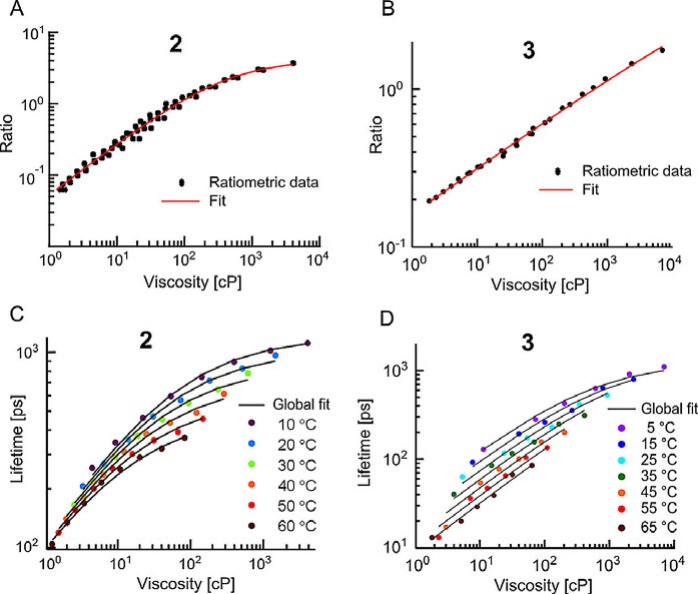
A method for sensing viscosity and temperature simultaneously with porphyrin dimers **2** and **3**. The ratios of the emission peaks of dimers with respect to viscosity are shown in A) dimer **2** and B) dimer **3** together with fits. C) and D) show the temperature/viscosity dependence of the lifetimes of the “twisted” conformers of **2** (C) and **3** (D). The lifetime data were globally fitted using equations derived according to photophysical models shown Figure 6. (C) was reproduced from Ref. 25 with permission from the Royal Society of Chemistry.

The twisted/planar ratios of porphyrin dimers **2** and **3** (Figure 2) are sensitive to viscosity only, thus the ratios recorded in an unknown environment can be used to obtain the viscosity values in the vicinity of the rotor. These temperature‐ independent ratiometric viscosity calibration curves (with fits) for both dimers are shown in Figure 5 A, B. The function used for fitting was derived in our previous work^18^ and has the following form [Eq. (1)]:

(1) $$r=\frac{1}{a_{1}\eta^{a_{2}}+a_{3}}$$

in which *r* is the ratio, *η* viscosity and *a*_1_, *a*_2_, and *a*_3_ are fitting parameters. The parameters obtained for the fits are shown in Table S1 (Supporting Information) While the data for **3** can also be fitted with a linear function, we use the same function in both cases to compare the fitting parameters.

In contrast to the twisted/planar peak ratios, the lifetimes of both dimers are affected by both temperature and viscosity (Figure 4). The lifetime data for **2** can be globally fitted (Figure 5 C) as demonstrated in our previous work^25^ using the following function [Eq. (2)]:

(2) $$\tau =\frac{1}{a_{1}\eta^{a_{2}}+a_{3}e^{-a_{4}/T}+a_{5}}$$

in which *τ* is lifetime, *η* viscosity, *T* temperature and *a*_1_, *a*_2_, *a*_3_, *a*_4_ and *a*_5_ are fitting parameters. Equation (2) provides the relationship between the lifetime and two unknown parameters: viscosity and temperature. Since viscosity can be measured by ratiometric measurements without any temperature bias, simultaneously performing both the lifetime and the ratiometric measurements allows us to measure viscosity and temperature simultaneously, for which both parameters can be extracted by applying Equations (1) and (2).

**Figure 6:**
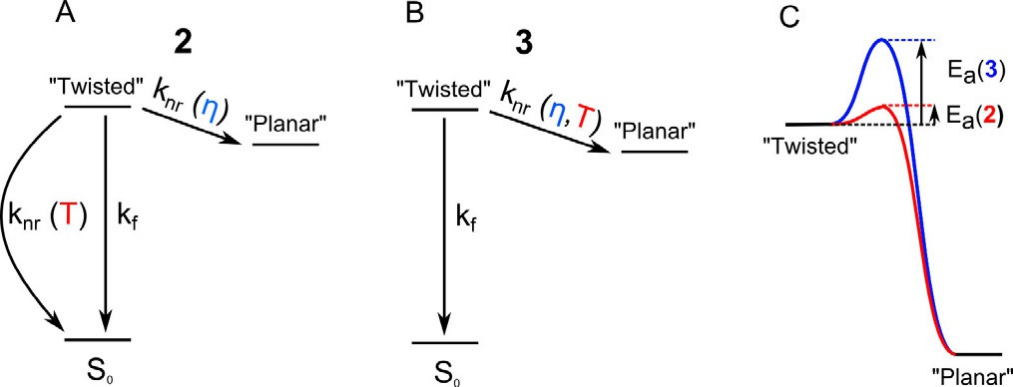
Photophysical models for dimers **2** (A) and **3** (B), which were used to derive functions for global fitting of the lifetime data in Figure 5. C) A suggested energy diagram for the “twisted”‐to‐“planar” transition showing different activation energies for dimers **2** and **3**. Note, [Eq. 2] assumes that *E*_a_(**2**)=0.

Equation (2) was derived previously,^25^ according to the photophysical model shown in Figure 6 A. This model assumes that there are three main de‐excitation pathways for an excited “twisted” state: i) radiative deactivation with a rate constant *k*_f_, ii) non‐radiative decay due to intramolecular twisting to form the “planar” state with a rate constant *k*_nr_(*η*)=*a*_1_ *η* ${}^{a{}_{2}}$ according to the Förster‐Hoffmann equation, and iii) non‐radiative decay sensitive to temperature with a rate constant *k*_nr_(*T*)= *a*_3_ *e* ${}^{-a{}_{4}/T}$ , an Arrhenius‐type term. Equation (2) was successfully used to globally fit the temperature‐dependent lifetime data for **2** (Figure 5 C), and the fitting parameters are shown in Table S1 (Supporting Information). The presence of the temperature‐dependent non‐radiative decay pathway effectively sets a limit on the maximum lifetime achievable at infinitely high viscosity, and this limiting lifetime decreases with increasing temperature. This is indeed observed in the data for dimer **2** (Figure 5 C); each isothermal curve asymptotically approaches its maximally possible lifetime value, which appears to decrease with temperature. The activation energy of the temperature‐dependent pathway can be estimated from the fitting parameter *a*_4_ (Table S1); *a*_4_ is equivalent to *E*_a_/*R* in the Arrhenius equation, in which *R* is the gas constant (8.314 J K mol^−1^). From this, *E*_a_=*R* *a*_4_=22 kJ mol^−1^.

Interestingly, we were not able to fit the lifetime data of dimer **3** (Figure 5 D) using Equation (2). As can be seem in Figure 5, while the lifetimes observed for dimer **2** converge to one point at low viscosities (<10 cP); this is not the case for the lifetimes of dimer **3**. We concluded that the photophysical model proposed for dimer **2** does not apply for dimer **3**.

We suggested that the intramolecular rotation in **3** may be affected by both viscosity and temperature. This is likely to be the case if a significant activation energy barrier is present for the “twisted”‐to‐“planar” conversion. In this case, the rate of isomerisation can be expressed as Equation (3):

(3) $$k_{i}=A\left(\eta \right)e^{-E_{a}/kT}$$

in which *k*_i_ is the rate, *E*_a_ is the energy of activation, *k* is the Boltzmann constant and *T* is temperature. According to Equation (3), both viscosity and temperature affect the conversion rate by determining the frequency (via *A*(*η*)) and the likelihood (via the Arrhenius‐type term). We substituted the Förster– Hoffmann term *a*_1_ *η* ${}^{a{}_{2}}$ for *A*(*η*) in Equation (3) to obtain a more generalised expression for the rate of the non‐radiative decay due to “twisted′‐to‐”planar“ transition in Equation (4):

(4) $$k_{nr}=a_{1}\eta^{a_{2}}e^{-a3/T}$$

For simplicity, to fit the lifetime data for dimer **3,** we assume that only two decay pathways for the “twisted” conformer exist: i) the radiative decay pathway, with a rate constant *k*_f_ and ii) the “twisted”‐to‐“planar” transition with rate constant *k*_nr_(*η*, *T*). The corresponding photophysical Scheme is shown in Figure 6 and the expression for the temperature/viscosity effect on the lifetime of **3** can be seen in Equation (5):

(5) $$\tau =\frac{1}{k_{\mathrm{nr}}\left(\eta ,T\right)+k_{f}}$$

Combining Equations (4) and (5), leads to Equation (6), which can be used to fit the lifetime data of dimer **3**:

(6) $$\tau =\frac{1}{a_{1}\eta^{a_{2}}e^{-a_{3}/T}+a_{4}}$$

Equation (6) was successfully used to fit the temperature‐ and viscosity‐dependent lifetime data for dimer **3** (Figure 5 D, parameters are shown in Table S1 in the Supporting Information). A good global fit of this data supports our predicted photophysical model for dimer **3**. The activation energy for the “twisted”‐to‐“planar” transition for dimer **3** can be calculated using the value of parameter *a*_3_ (*E*_a_=*R* *a*_3_; 18 kJ mol^−1^). We have attempted to include an additional term *a*_5_ *e* ${}^{-a{}_{6}/T}$ in Equation (6), which should account for the temperature‐dependent deactivation pathway similar to that present for dimer **2**, however this additional term did not improve the fit (see details in the Supporting Information, Figure S5). We note that the pathway described by *a*_5_ *e* ${}^{-a{}_{6}/T}$ might still be present for dimer **3**, however, it is not required to successfully fit the effects of viscosity or temperature on the lifetime of **3**.

The lifetimes of the planar conformer of dimer **3** are also sensitive to temperature, (Figure S6), which allows us to use the full spectral range of **3** for viscosity/temperature dual measurements.

Thus, both dimers **2** and **3** can be used for sensing viscosity and lifetime simultaneously by combined ratiometric and lifetime measurements, albeit by utilising different mechanisms of sensitivity to temperature. We hypothesise that the main reason for the intriguing difference in responses of dimers **2** and **3** to viscosity and temperature is the difference in activation energies for “twisted”‐to‐“planar” transitions in both dimers (Figure 6 C). For dimer **2**, activation energy is negligible, which makes the rate of intramolecular twisting independent of temperature. As can be seen from Figure 5 C, the temperature dependence of lifetimes is significant at high viscosities (>50 cP), only when the temperature‐dependent rate *k*_nr_(*T*) becomes the dominant deactivation rate. On the other hand, the activation energy for dimer **3** is significant, which makes the rate of twisting both viscosity‐ and temperature‐dependent and this leads to a large difference in lifetimes at different temperatures at all viscosities (Figure 5 D).

Interestingly, dimer **2** is more suitable for measurements in lower viscosity (<100 cP) and higher temperature (>40 °C) environments due to its lifetime range. Namely, all recorded lifetimes of dimer **2** are above 100 ps, which can be reliably measured by most time‐correlated single photon counting (TCSPC) setups.

However, at high viscosities (>100 cP) and low temperatures (<40 °C) the lifetime of dimer **2** becomes less sensitive to viscosity due to a reduced log(lifetime)/log(viscosity) slope in the Förster–Hoffmann calibration. This is precisely the range in which dimer **3** becomes more useful (Figure 4). Additionally, dimer **3** shows no saturation in the log(lifetime)/log(viscosity) plot. Therefore, dimers **2** and **3** complement each other as dual viscosity and temperature sensors for low and high viscosity environments.

**Figure 7:**
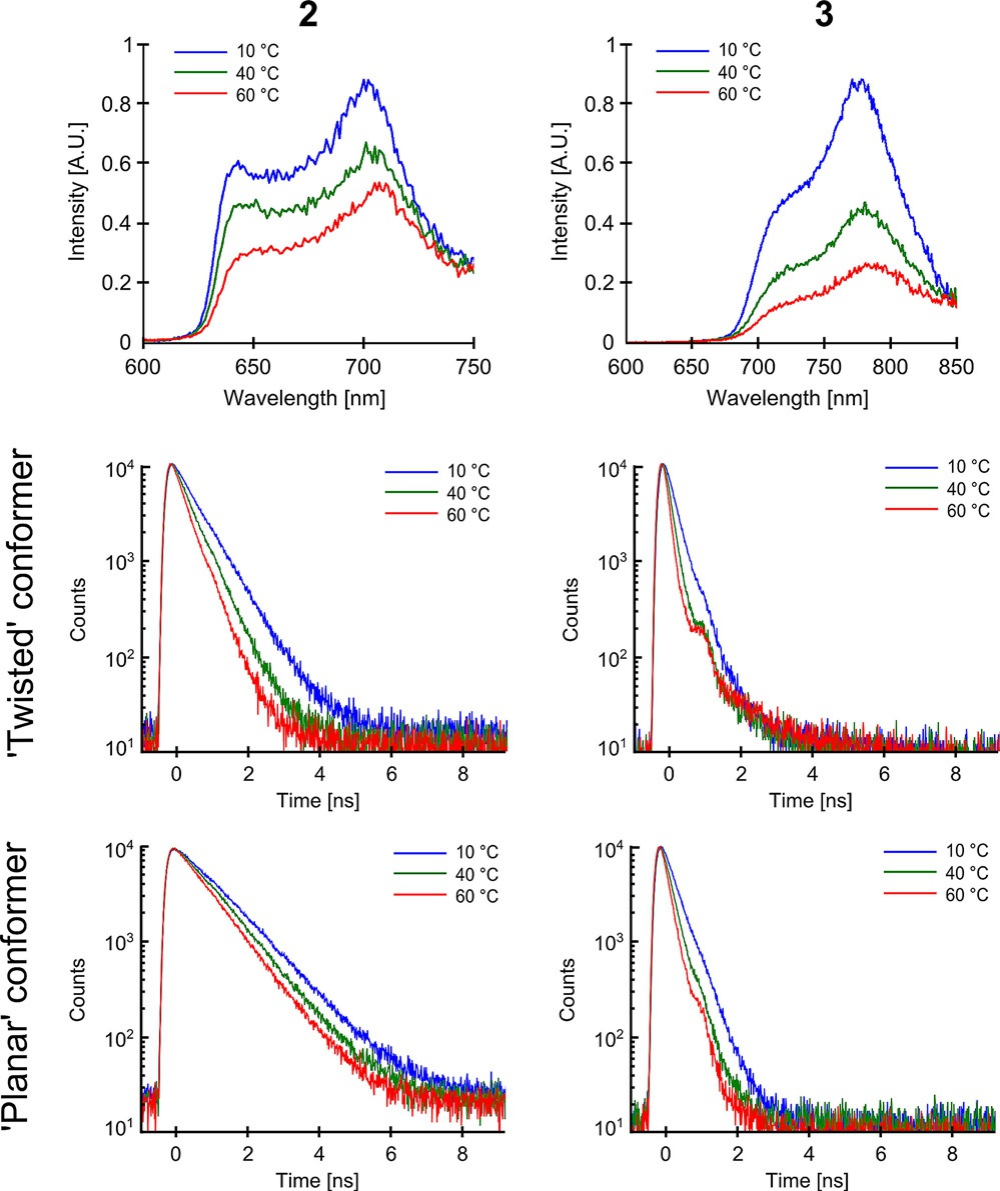
Fluorescence spectra and fluorescence decays of **2** (left column) and **3** (right column) in 6:4 (*v*/*v*) glycerol/methanol at 10 °C, 8:2 glycerol/methanol at 40 °C and 9:1 glycerol/methanol at 60 °C. Viscosities of the mixtures were 54, 52 and 40 cP, respectively. The spectra are shown in the top row. The fluorescence decays of the “twisted” and the “planar” conformers of both dimers are shown in the middle and the bottom rows, respectively. The spectra and the decays were recorded upon 453 nm (**2**) and 473 nm (**3**) excitation. The decays of **2** were recorded at 640 nm (“twisted”) and 700 nm (“planar”); the decays of **3** at 720 nm (“twisted”) and 780 nm (“planar”); 10 nm bandwidth in all cases.

To further confirm the intriguing temperature dependence of the time resolved decays of dimers **2** and **3**, we measured fluorescence spectra of both dimers in glycerol and methanol mixtures of different composition at different temperature, but at matching viscosities (Figure 7). It can be clearly seen that the intensity of the fluorescence spectra decreases with increasing temperature, but the shape of the spectra does not change and the ratio of the peaks is preserved. Fluorescence decay rates of the “twisted” and the “planar” conformers of **2** and **3** (Figure 7) both increase at higher temperatures. Therefore, higher temperature accelerates the non‐radiative decay rate back to the ground state, which equally affects both conformers and leaves the fluorescence spectrum unchanged. We also note that the decay rate of the “planar” conformer of **2** changes with increasing temperature to a lesser extent than the decay rate of the “twisted” conformer. We infer that the formation of the “planar” state (presumably from the “twisted” state) is reduced at higher temperatures, which is an additional factor contributing to the overall lower intensity of the “planar” peak in the spectrum. While the reasons for the unusual temperature dependence of the time‐resolved fluorescence decays of **2** and **3** compared to **1** and **4** are not entirely clear, we hypothesise that it may originate from viscosity‐ dependent rotation of phenyl rings at the periphery of these molecules, similar to what was previously implemented in the decrease in fluorescence of rhodamine B in alcohols at increasing temperature.^37^

## Conclusion

In conclusion, we have analysed and contrasted the fluorescence properties of four porphyrin dimers, uniquely constructed for sensing of viscosity by ratiometric or lifetime‐based methods, in different viscosity environments at different temperatures. It should be noted that the majority of ratiometric viscosity probes are designed by fusing two independent fluorophores together, only one of which is a rotor.^3^, ^38^ There are only a few reports of single fluorophores (apart from conjugated porphyrin dimers) with complex excited state interplay leading to ratiometric sensitivity towards viscosity.^30^, ^39^

The photophysical properties along with advantageous/recommended use for each porphyrin dimer are summarised in Table 1. Porphyrin dimers **1** and **4**, which were not investigated as viscosity sensors previously, are shown to be promising dual‐viscosity sensors capable of measuring viscosity both by lifetime and ratiometric methods in low (**1**) and moderate (**4**) polarity environments. The previously reported far‐red emitting ratiometric viscosity sensor **3** is shown to be suitable as lifetime‐based viscosity sensor for higher viscosity (>100 cP) environments with advantages over the previously reported dimer **2**. A particular advantage of **3** is the ability to perform measurements in extremely far‐red spectral range (>700 nm), due to the involvement of both the planar and the twisted conformers in the viscosity‐dependent deactivation, at least following one‐photon excitation. Dimers **2** and **3** were found to display unique dual temperature and viscosity sensitive lifetimes, in which temperature‐sensitivity occurs by two different mechanisms. Consequently **2** and **3** can be used as dual viscosity and temperature probes, with dimer **2** giving advantages in measurements in lower viscosity (<100 cP) and higher temperature (>40 °C) environments, while dimer **3** is more suitable for a high viscosity environment, due to its lifetime range. Overall, porphyrin dimers are a fascinating family of fluorescent compounds that can prove useful for measuring microscopic viscosity and temperature, unavailable with any other currently available fluorescent dyes.

**Table 1:** Photophysical parameters of porphyrin dimers and their advantages.

Porphyrin dimer	“Twisted” absorption maximum [nm]	“Twisted” fluorescence maximum [nm]	“Planar” fluorescence maximum [nm]	Sensitive to temperature?	Advantages
**1**	450	635	690	No	Soluble in non‐polar environments. Dual ratio/lifetime viscosity sensor.
**2**	453	640	700	Yes	Viscosity and temperature sensor. Particularly sensitive in the low‐to‐moderate viscosity range.^[a]^
**3**	473	710	780	Yes	The most red‐shifted fluorescence spectrum.^[b]^ Viscosity and temperature sensor. More sensitive in high viscosity, low temperature environments^[c]^. Both conformers are viscosity and temperature sensitive.
**4**	450	650	710	No	Suitable for hydrophilic environments. Dual ratio/lifetime viscosity sensor.

[a] 1–100 cP. [b] Completely in the tissue optical window. [c]>100 cP, <40 °C.

## Experimental Section

### Porphyrin dimers and solvents

Porphyrin dimers **1**–**3** were synthesised as reported previously.^40^ The synthesis and characterisation of dimer **4** is detailed in the Supporting Information. For spectroscopic measurements, 1 mm stock solutions of all dimers were prepared in dimethylsulfoxide and diluted into the solvent of interest (≈0.1 % *v*/*v* of DMSO in each mixture). DMSO, methanol, toluene and castor oil (all of spectroscopic grade) were obtained from Sigma Aldrich. Spectroscopic grade glycerol was obtained from Alfa‐Aesar. Methanol/glycerol mixtures at variable temperatures were used for the calibration of dimers **2**, **3** and **4**. The spectroscopic measurements for the hydrophobic dimer **1** were performed in toluene/Castor oil mixtures. Viscosities of all mixtures were measured with a Stabinger viscometer (SVM3000, Anton Paar) with ±0.35 % accuracy. The small quantity of DMSO present was confirmed not to affect the viscosities of the mixtures.

### Absorption and fluorescence spectra

Absorption spectra were measured using an Agilent 8453 UV/Vis spectrophotometer. A Fluoromax‐4 spectrofluorometer (Jobin–Yvon, Horiba) was used for measuring fluorescence spectra of dimers **2**, **3** and **4**, fluorescence spectra of dimer **1** were measured by an Ocean Optics QE 6500 spectrometer. The temperature of cuvette in the fluorometer was controlled by a Peltier thermostated cuvette holder (F3004, Jobin–Yvon, Horiba) to 0.5 °C accuracy. Quartz cuvettes with 10 mm path length were used. The concentration of the dimers for absorption and fluorescence measurements was 0.33 μm leading to absorbance of ≈0.1.

### Time‐resolved fluorescence

Fluorescence decays of porphyrin dimers were measured using a time‐correlated single photon counting (TCSPC) setup. A coherent Chameleon Vision II mode‐locked femtosecond Ti:sapphire laser was used as excitation source together with a second harmonic generator (Harmonic, Coherent) for frequency doubling. The laser produced pulses of 140 fs with temporal width at 80 MHz and frequency at 680–1080 nm wavelength range, which can be frequency doubled to 340–540 nm. Excitation power was no more than 1 mW for one‐photon excitation. The cuvette holder (qpod, Quantum Northwest) was temperature‐controlled by a Peltier device (TC 125, Quantum Northwest). Emission wavelength was selected by an Omni‐*λ* 150 (LOT‐Quantum Design) grating monochromator. The detector was based on a DCC‐100 (Becker & Hickl) detector control module with a PMC‐100‐1 (Hamamatsu) photomultiplier tube connected to a SPC‐830 TCSPC card (Becker & Hickl). Fluorescence decays of dimer **3** over the 680–800 nm range were measured using a FLIM system based on a Leica SP5‐II microscope and the above‐mentioned laser. The time window size for fluorescence decays was 12.5 ns, which was split into 1024 channels. An instrument response function (IRF) was measured by recording scattering from a cuvette containing a Ludox® solution. Both fluorescence decays and spectra of porphyrin dimers were measured in mixtures covering a 1–1000 cP viscosity range in the 5–85 °C temperature range.

### Data analysis

Fitting of fluorescence decays was done using a home‐written code on MATLAB R2012a. Further data processing and analysis was done with OriginPro 8.6.

## Conflict of interest

The authors declare no conflict of interest.

## Supporting information

As a service to our authors and readers, this journal provides supporting information supplied by the authors. Such materials are peer reviewed and may be re‐organized for online delivery, but are not copy‐edited or typeset. Technical support issues arising from supporting information (other than missing files) should be addressed to the authors.

Supplementary
