# Supplementary material for: Tuning the Sensitivity of Fluorescent Porphyrin Dimers to Viscosity and Temperature
Source: Chemistry. 2017 Jun 13;23(46):11001–10. doi: 10.1002/chem.201700740 (PMC5575558; doi:10.1002/chem.201700740)
Supplement: Supplementary file 1 — Supplementary [file CHEM-23-11001-s001.pdf]

CHEMISTRY
A European Journal

Supporting Information

Tuning the Sensitivity of Fluorescent Porphyrin Dimers to Viscosity and Temperature

Aurimas Vyšniauskas,[a] Dong Ding,[a] Maryam Qurashi,[a] Igor Boczarow,[b] Milan Balaz,[b, c] Harry L. Anderson,[b] and Marina K. Kuimova*[a]

chem_201700740_sm_miscellaneous_information.pdf

Table of Contents

1. Synthesis S2
2. Additional data S4
    Figure S1 .... S4
    Figure S2 .... S5
    Figure S3 .... S6
    Figure S4 .... S6
    Table S1 .... S6
    Figure S5 .... S7
    Figure S6 .... S7
3. Future directions for the probe design S8
4. Bibliography S8

1. Synthesis

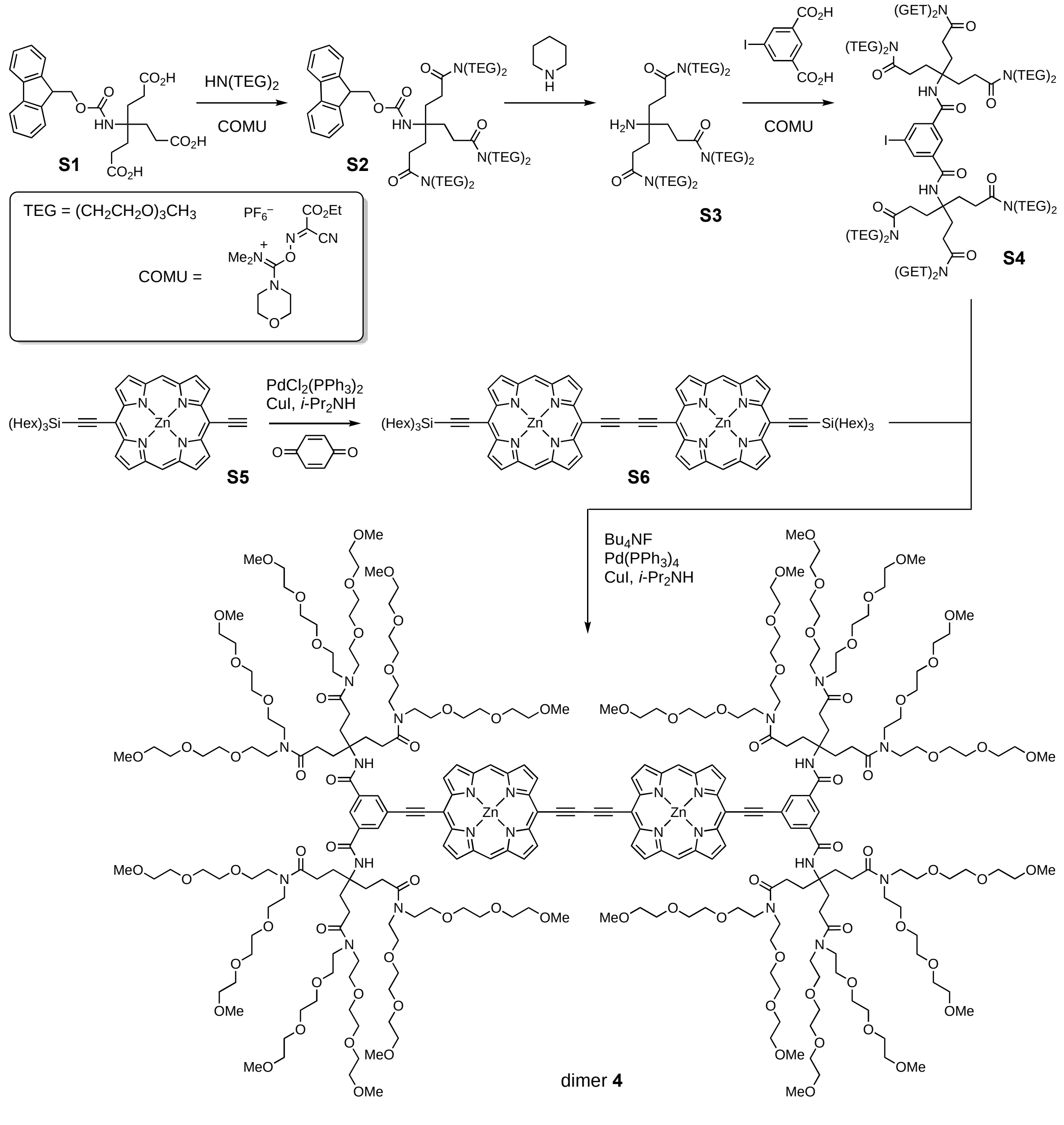

Scheme S1. Synthesis of dimer **4**.

Procedure for Synthesis of Protected Amine S2. Compound **S1** (50 mg, 107 µmol) was dissolved in a mixture of DMF (0.5 mL) and DIPEA (0.11 mL, 1.3 mmol), and cooled to 0 °C. The COMU coupling reagent (175 mg, 640 µmol) was added and the mixture was stirred for 1 min at 0 °C, before adding a solution of $HN(TEG)_2$ [1] (200 mg, 604 µmol) in DMF (0.5 mL) and DIPEA (0.11 mL, 1.3 mmol) at 0 °C. The mixture was stirred at 0 °C for 1 h, then at room temperature for 2 h. The crude reaction mixture was diluted with EtOAc (20 mL) and washed with HCl (1 M, 2 x 5 mL), $NaHCO_3$ (1 M aq., 2 x 5 mL) and saturated NaCl aq. (2 x 5 mL). The aqueous phase was additionally washed with CH_2Cl_2 (4 x 150 mL) then combined with the organic phase. The product was purified by size exclusion chromatography (Bio-Beads® SX-1, 200–400 mesh; $CHCl_3$) to yield **S2** as a yellow oil (100 mg, 70 %). ^{1}H NMR (400 MHz, $CDCl_3$) δ = 7.73 (d, 2H, *J* = 7.6 Hz, Ar-CH), 7.60 (d, 2H, *J* = 7.5 Hz, Ar-CH), 7.36 (t, 2H, *J* = 7.4 Hz, Ar-CH), 7.27 (t, 2H, *J* = 7.6 Hz, Ar-CH), 6.61 (s, 1H, NH), 4.22 (m, 2H, CH_2), 4.17 (m, H,

CH), 3.64–3.44 (m, 72H, TEG-CH_2), 3.33 (s, 9H, TEG-CH_3), 3.31 (s, 9H, TEG-CH_3), 2.41 (t, 6H, *J* = 7.0 Hz), 2.01 (t, 6H, *J* = 6.7 Hz) ppm. *m/z* (MALDI-ToF) 1366.72 ($[M+Na]^+$ 100%, $C_{67}H_{144}N_4O_{23}Na^+$ requires 1365.78).

Procedure for Synthesis of Amine S3. Compound **S2** (200 mg, 14.9 μmol) was stirred with piperidine (3 mL) in DMF (12 mL) for 1 h at room temperature. The solvent was removed by evaporation and the residue was purified by chromatography on SiO_2 (eluting with $CHCl_3$: MeOH 100:2 to 100:15), to yield **S3** as a yellow oil (153 mg, 90%). ^1H NMR (400 MHz, $CDCl_3$) δ = 3.55–3.40 (m, 72H, TEG-CH_2), 3.27 (s, 9H, CH_3), 3.26 (s, 9H, CH_3), 2.43 (t, 6H, *J* = 7.2 Hz, CH_2), 1.71 (t, 6H, *J* = 7.1 Hz, CH_2) ppm. *m/z* (MALDI-ToF) 1145.22 ($[M+Na]^+$ 100%, $C_{52}H_{104}N_4O_{21}Na^+$ requires 1143.71).

Procedure for Synthesis of Aryl Iodide S4. 3-Iodoisophthalic acid (50 mg, 67 μmol) was dissolved in a mixture of DMF (0.2 mL) and DIPEA (70 μL, 0.8 mmol), and cooled to 0 °C. The COMU coupling reagent (218 mg, 0.8 mmol) was added and the mixture was stirred for 1 min at 0 °C before adding a solution of compound **S3** (247 mg, 0.8 mmol) in DMF (0.2 mL) and DIPEA (70 μL, 0.8 mmol) at 0 °C. The mixture was stirred at 0 °C for 1 h, then at room temperature for 2 h. The crude reaction mixture was diluted with EtOAc (20 mL) and washed with HCl (1 M, 2 x 5 mL), $NaHCO_3$ (1 M aq., 2 x 5 mL) and saturated NaCl aq. (2 x 5 mL). The aqueous phase was additionally washed with CH_2Cl_2 (4 x 200 mL) then combined with the organic phase. The product was purified by size exclusion chromatography (Bio-Beads® SX-1, 200–400 mesh; $CHCl_3$) to yield **S4** as an oil (131 mg, yield 78%). ^1H NMR (400 MHz, $CDCl_3$) *δ* = 8.76 (broad s, 2H, amide-NH), 8.40 (s, 1H, Ar-H), 8.30 (s, 2H, Ar-H), 3.63–3.47 (broad m, 74H, TEG-CH_2), 3.35 (s, 9H, TEG-OCH_3), 3.33 (s, 9H, TEG-OCH_3), 2.46 (t, 6H, *J* = 6.2 Hz, CH_2), 2.15 (t, 6H, *J* = 6.2 Hz, CH_2) ppm. *m/z* (MALDI-ToF) 2521.92 ($[M+Na]^+$ 100%, $C_{112}H_{209}IN_8O_{44}Na^+$ requires 2521.33).

Procedure for Synthesis of Porphyrin Dimer S6. Porphyrin monomer **S5** (60 mg, 86 μmol)[2,3] was added to a solution of $PdCl_2(PPh_3)_2$ (4.0 mg, 5.8 μmol), CuI (7.6 mg, 2.9 μmol) and 1,4-benzoquinone (17.8 mg, 0.16 mmol) in THF (8 mL) and DIPA (2 mL). The mixture was stirred under N_2 at room temperature for 90 min. TLC (PET ether 40–60 °C : EtOAc : py, 10 : 1 : 1) indicated complete reaction. The mixture was passed through a silica plug ($CHCl_3$: pyridine, 100 : 1), concentrated and purified by recrystallization from $CHCl_3$:MeOH to yield porphyrin dimer **S6** as a dark green powder (38.4 mg, yield 64%). ^1H NMR (400 MHz, $CDCl_3$) *δ* = 10.07 (s, 4H, *meso*-CH), 10.04 (d, 4H, *J* = 4.2 Hz, β-CH), 9.80 (d, 4H, *J* = 4.4 Hz, β-CH), 9.39 (d, 4H, *J* = 4.4 Hz, β-CH), 9.31 (d, 4H, *J* = 4.4 Hz, β-CH), 1.86 (m, 12H, hexyl-CH_2), 1.63 (m, 12H, hexyl-CH_2), 1.47 (m, 24H, hexyl-CH_2), 1.10 (m, 12H, hexyl-CH_2), 0.97 (t, 18H, *J* = 6.9 Hz, hexyl-CH_3) ppm. *m/z* (MALDI-ToF) 1406.62 ($[M]^+$ 100%, $C_{84}H_{98}N_8Si_2^+$ requires 1406.60).

Procedure for Synthesis of Porphyrin Dimer 4. *n*-Bu_4NF (1.0 M in THF, 13 μL, 13 μmol) was added to a solution of zinc-porphyrin dimer **S6** (10 mg, 7.1 μmol), aryl iodide **S4** (35 mg, 14 μmol), $Pd(PPh_3)_4$ (1.6 mg, 1.4 μmol) and CuI (0.30 mg, 1.4 μmol) in THF (1.5 mL), DIPA (1.5 mL) and pyridine (34 μL) under an atmosphere of N_2. The mixture was stirred at 50 °C for 2.5 h. MALDI ToF MS analysis indicated that the reaction was complete. The crude reaction mixture was passed through a silica plug (THF, MeOH, 2 : 1), concentrated and purified by size-exclusion chromatography (Bio-Beads® SX-1, 200–400 mesh) to yield the dimer **4** as a green glass (20 mg, 50%). ^1H NMR (500 MHz, $CDCl_3$) *δ* = 10.10 (s, 4H,*meso*-CH), 10.02 (d, 4H, *J* = 4.4 Hz, β-CH), 9.85 (d, 4H, *J* = 4.3 Hz, β-CH), 9.40 (d, 4H, *J* = 4.5 Hz, β-CH), 9.32 (d, 4H, *J* = 4.5 Hz, β-CH), 8.82 (s, 4H, amide-NH), 8.63 (s, 4H, Ar-H), 8.57 (s, 4H, Ar-H), 3.60–3.28 (m, 296H, TEG(CH_2)-H), 3.21 (s, 36H, TEG(CH_3)-H), 3.20 (s, 36H, TEG(CH_3)-H), 2.52 (t, 24H, *J* = 6.2 Hz, CH_2), 2.24 (t, 24H, *J* = 6.3 Hz, CH2) ppm. *m/z* (MALDI-ToF) 5600.69 ($[M+H_2O]^+$ 100%, $C_{272}H_{439}N_{24}O_{89}Zn_2^+$ requires 5600.93).

2. Additional data

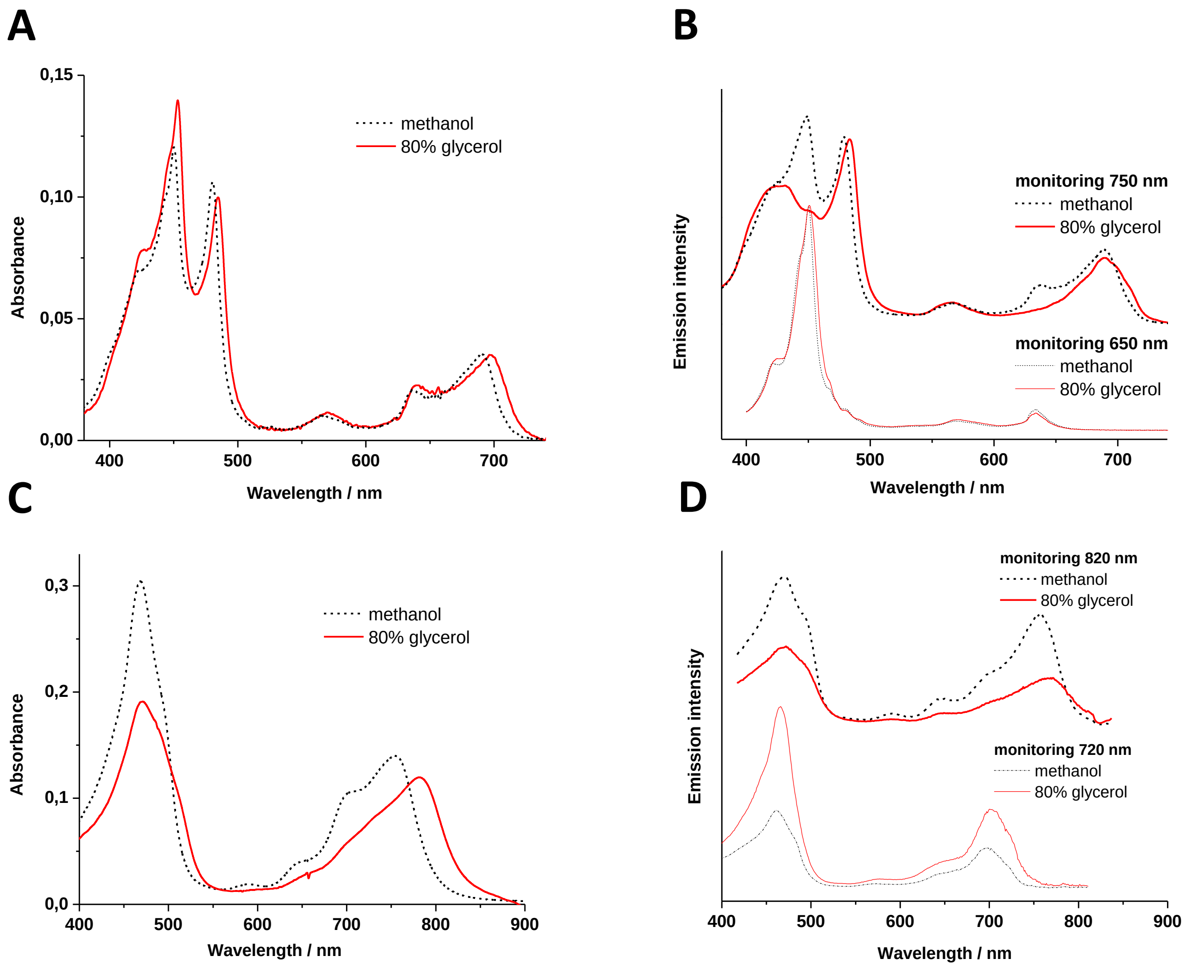

Figure S1. Absorption (A, C) and excitation (B, D) spectra of **2** (A, B) and **3** (C, D) in methanol (--) and 1:4 methanol-glycerol (v/v) (red solid line). Excitation spectra were obtained at the maxima for the twisted conformers (650 nm in B and 720 nm in D) and the planar conformers (750 nm in B and 820 nm in D). The peaks in the excitation spectra show a minimal shift with increasing viscosity.

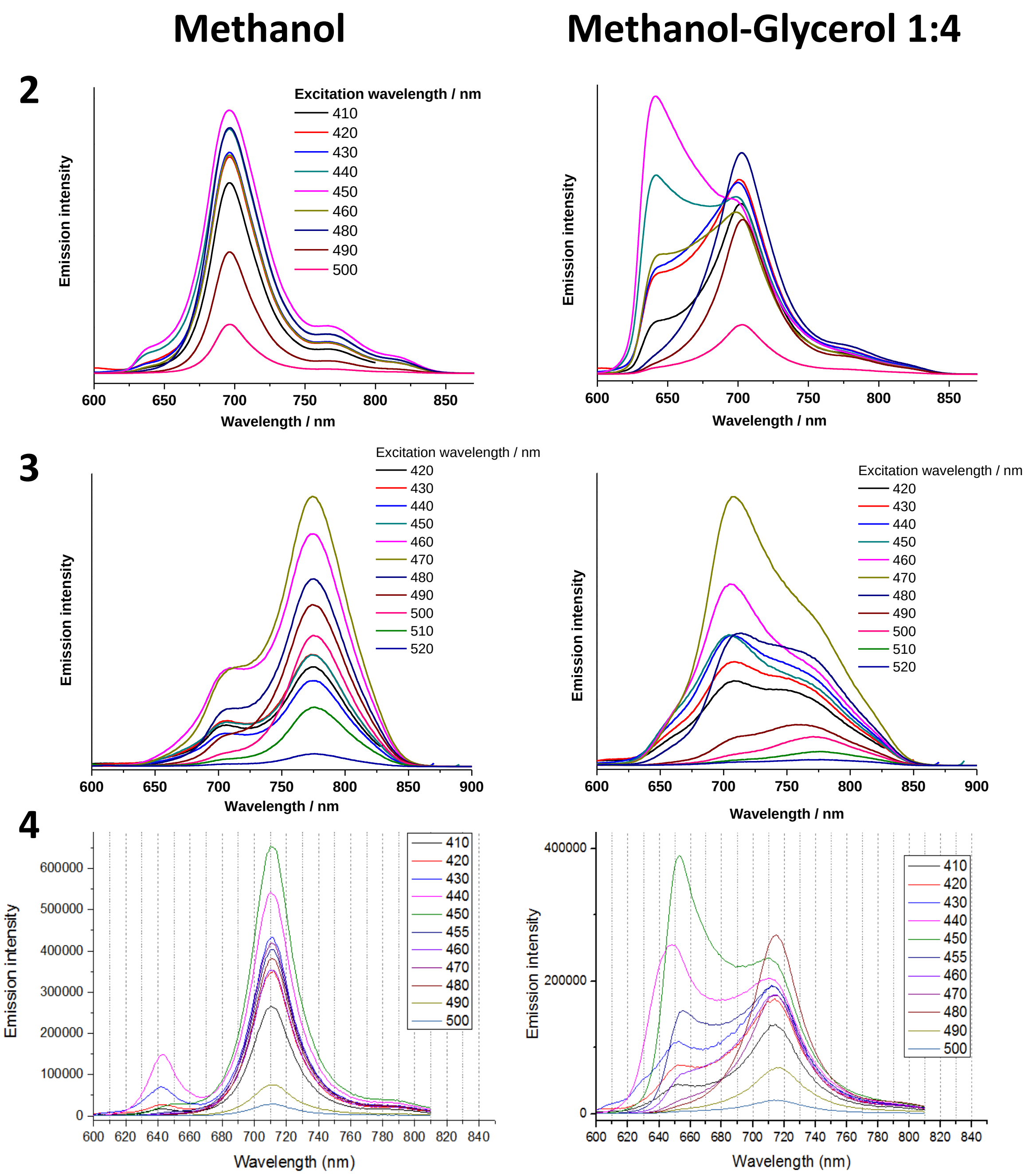

Figure S2. Emission spectra of **2** (top row), **3** (middle row) and **4** (bottom row) in methanol (left column) and 1:4 methanol-glycerol (v/v) (right column), following excitation at wavelengths over the range 400-520 nm.

It could be seen that the excitation into the long-wavelength component of the B band for dimers **2** and **4** results mainly (but not exclusively) in low energy fluorescence, i.e. from the low-energy (planar) component. However, a small fluorescence intensity from the twisted band can be detected, particularly in viscous solutions, due to a small overlap between the B bands of the planar and the twisted components (see Figure S1).

On the other hand, for **3** the overlap between the excitation spectra of the twisted and the planar conformers is very strong and, therefore, both conformers are excited at all wavelengths.

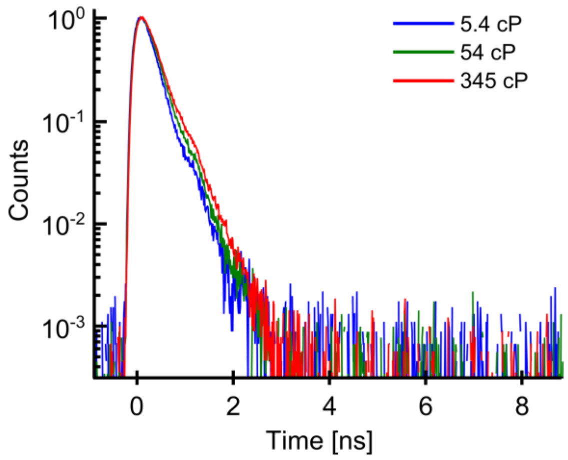

Figure S3. Two-photon excited (900 nm) normalised fluorescence decays of the 'planar' conformer of dimer **3** at 780 nm. The decays have at least 5 000 counts at the peak. Fluorescence decays are nearly independent on viscosity contrasting the data obtained upon one photon excitation (Figure 3, main text).

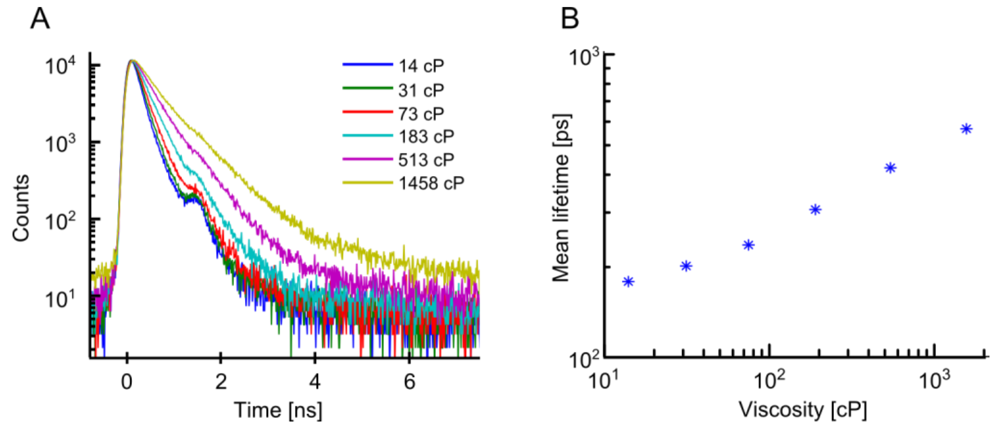

Figure S4. A) Fluorescence decays of dimer **3** in glycerol and methanol mixtures at 20 ˚C collected over its full fluorescence spectrum, from 680 to 800 nm. B) Intensity-weighted mean lifetimes of decays shown in a). The decays were fitted using biexponential function. Excitation wavelength was 473 nm.

Table S1. Parameters of the fits shown in Figure 5, main text.

	Porphyrin dimer	a_1	a_2	a_3	a_4	a_5
**Ratiometric fits**	**2**	19	-0.73	0.24	-	-
	**3**	6.1	-0.30	0.09	-	-
**Global lifetime fits**	**2**	$8.1\text{x}\times10^{-3}$	-0.58	4.7	$2.6\text{x}\times10^{3}$	$4.3\text{x}\times10^{-4}$
	**3**	79	-0.56	$2.2\text{x}\times10^{3}$	$8.1\text{x}\times10^{-4}$	-

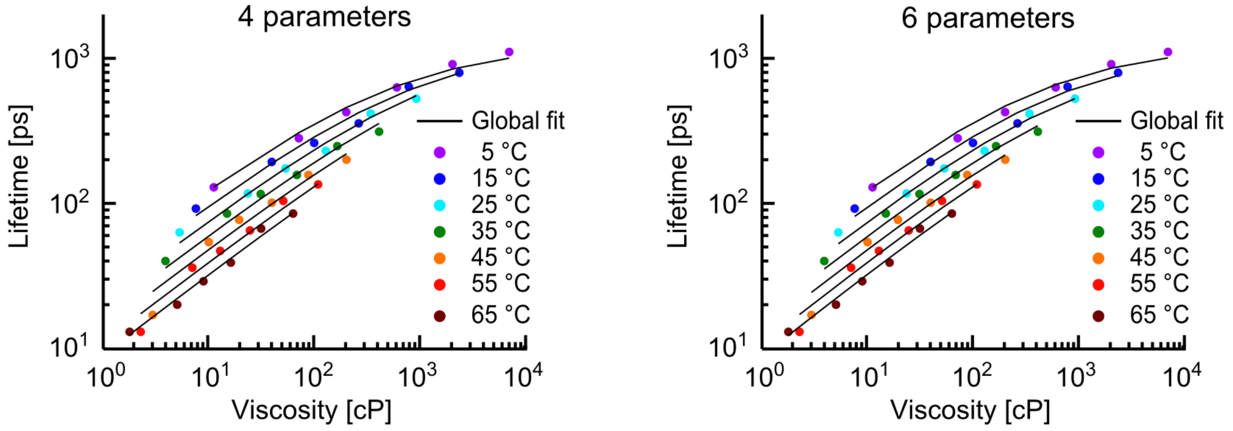

Figure S5. The comparison of global fits used to fit the lifetime data of dimer **3**. The fit on the left was obtained using Equation 6 (main text), which contains 4 free parameters. Fitting was also attempted after expanding Equation 6 with an additional $a_5 e^{-a_6/T}$ term, which accounts for the temperature-dependent deactivation path shown to be present for dimer **2**. The resulting fit is shown on the right, which is almost identical to the one on the left. The only difference is slightly better-fitted data points at high viscosities at 15, 25 and 35 ˚C. However this is a marginal improvement only. As a result, Equation 6 was used without the additional term to fit the data of dimer **3**.

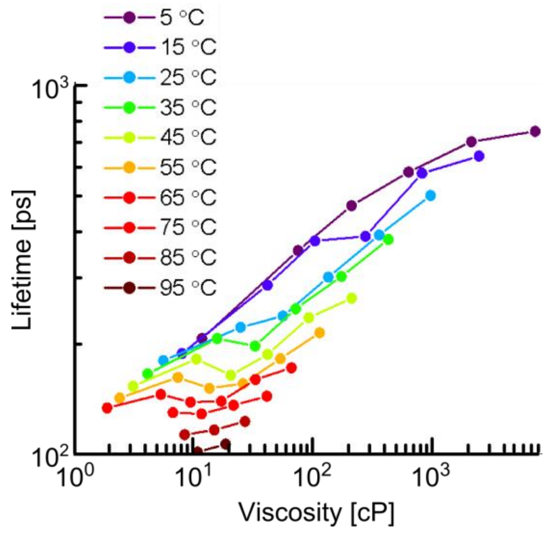

Figure S6. The fitted lifetimes of the planar conformer of dimer **3** measured at 780 nm upon 473 nm excitation in glycerol and methanol mixtures at a range of temperatures, as shown in the legend.

3. Future directions for the probe design

A slightly further hindrance of planarization might push the dynamic range of porphyrin dimers towards lower viscosities, due to the fact that viscosity-mediated ‘twisted’-to’-‘planar’ conversion in the excited state would be more difficult (will have a higher activation barrier). This hindrance can be achieved either sterically or electronically.

Sterically: An increase of bulk in substituents R_2 might hinder twisting, since a larger molecule should experience a stronger resistance to intramolecular rotation, especially in a viscous solvent. However, derivatives with bulky R_2s may be unsuitable for biological studies due to their poor penetration through cellular membranes.

Electronically: our current dimers have no TICT states, and the main driving force for the planarization of porphyrin dimers in the excited state is the stabilization of the singlet excited state, which has more cumulenic character of the central butadiyne bridge than the ground state. In the future, a range of dimers with various R_1s (donating or accepting), can be examined, in an attempt to tune the viscosity and temperature sensitivity. Only electron-accepting substituents have been examined by us in the present work and we observed the rotor behavior for all these molecules. However, it is possible that subtle changes in R_1 can be responsible for different temperature sensitivity in dimers **2** and **3**, observed in this work.

In the case of both a steric and an electronic tuning of the dimer properties, care should be taken:

(i) The increased height of the twisted-to-planar conversion barrier can result in different viscosity and temperature sensitivity, for example as observed for **3** versus **2** in this work (we have demonstrated that **3** has a higher activation barrier).

(ii) A strong hindrance would completely prevent a twisted to planar conformational change and, as a result, the molecule can lose all its sensing properties, similarly to what was reported recently.[4]

4. Bibliography

1. Selve, C. *et al.* Monodisperse perfluoro-polyethoxylated amphiphilic compounds with two-chain polar head - preparation and properties. *Tetrahedron* **47,** 411–428 (1991).

2. Reeve, J. E. *et al.* Amphiphilic porphyrins for second harmonic generation imaging. *J. Am. Chem. Soc.* **131,** 2758–9 (2009).

3. López-Duarte, I. *et al.* ‘Push-no-pull’ porphyrins for second harmonic generation imaging. *Chem. Sci.* **4,** 2024 (2013).

4. M. Macchione, N. Chuard, N. Sakai, S. Matile, *Chempluschem* **2017**, *82*, 1–6.
